# Views of Young People in Rural Australia on SPARX, a Fantasy World Developed for New Zealand Youth With Depression

**DOI:** 10.2196/games.3183

**Published:** 2014-02-18

**Authors:** Colleen Cheek, Heather Bridgman, Theresa Fleming, Elizabeth Cummings, Leonie Ellis, Mathijs FG Lucassen, Matthew Shepherd, Timothy Skinner

**Affiliations:** ^1^Rural Clinical SchoolUniversity of TasmaniaBurnieAustralia; ^2^Department of Rural HealthUniversity of TasmaniaLauncestonAustralia; ^3^Werry Centre for Child and Adolescent Mental healthDepartment of Psychological MedicineUniversity of AucklandAucklandNew Zealand; ^4^School of Nursing and MidwiferyUniversity of TasmaniaHobartAustralia; ^5^School of Computing and Information SystemsUniversity of TasmaniaHobartAustralia; ^6^School of CounsellingHuman Services and Social WorkUniversity of AucklandAucklandNew Zealand; ^7^School of Psychological and Clinical SciencesCharles Darwin UniversityDarwinAustralia

**Keywords:** mental health, stigma, computer games, youth, rural health, computerized CBT

## Abstract

**Background:**

A randomized control trial demonstrated that a computerized cognitive behavioral therapy (cCBT) program (Smart, Positive, Active, Realistic, X-factor thoughts [SPARX]) was an appealing and efficacious treatment for depression for adolescents in New Zealand. Little is known about the acceptability of computerized therapy programs for rural Australians and the suitability of computerized programs developed in one cultural context when used in another country. Issues such as accents and local differences in health care access might mean adjustments to programs are required.

**Objective:**

This study sought to explore the acceptability of SPARX by youth in rural Australia and to explore whether and how young people would wish to access such a program.

**Methods:**

Focus groups and semistructured interviews were conducted with 16 young people attending two youth-focused community services in a small, rural Tasmanian town. An inductive data-driven approach was used to identify themes using the interview transcripts as the primary data source. Interpretation was supported by demographic data, observer notes, and content analysis.

**Results:**

Participants reported that young people want help for mental health issues but they have an even stronger need for controlling how they access services. In particular, they considered protecting their privacy in their small community to be paramount. Participants thought computerized therapy was a promising way to increase access to treatment for youth in rural and remote areas if offered with or without therapist support and via settings other than school. The design features of SPARX that were perceived to be useful, included the narrative structure of the program, the use of different characters, the personalization of an avatar, “socialization” with the Guide character, optional journaling, and the use of encouraging feedback. Participants did not consider (New Zealand) accents off-putting. Young people believed the SPARX program would appeal to those who play computer games generally, but may be less appealing for those who do not.

**Conclusions:**

The findings suggest that computerized therapy offered in ways that support privacy and choice can improve access to treatment for rural youth. Foreign accents and style may not be off-putting to teenage users when the program uses a playful fantasy genre, as it is consistent with their expectation of fantasy worlds, and it is in a medium with which they already have a level of competence. Rather, issues of engaging design and confidential access appeared to be more important. These findings suggest a proven tool once formally assessed at a local level can be adopted cross-nationally.

##  Introduction

Northwest Tasmania is characterized by mountainous terrain, a rugged coastline, and small, sparsely populated settlements with economies reliant on agriculture, mining, and forestry. These communities are too small to individually host a full range of community health services and in winter they are often isolated due to flooding, high winds, snow fall, and icy roads. It is these smaller towns and settlements where social determinants associated with health inequity are most evident, that have reduced access to health services, have a higher proportion of Aboriginal Australians, lower socioeconomic status (SES), and are under-represented in post school education statistics [1,2]. Recent investment in Internet infrastructure in the region has provided an opportunity to support communities and augment existing services with Web-based interventions.

The impact of child and adolescent mental health disorders on individuals, families, and communities is significant [3,4]. Loss of engagement at school, increased substance abuse, family conflict, and teenage pregnancy are common comorbid associations [4-6]. Early intervention with cognitive behavior therapy (CBT) for young people with depression has been shown to be effective in reducing mental health symptoms [6-9]. Tasmania has the second highest risk of suicide of all Australian states, with an apparent urban-rural gradient for males (which places rural males at increased risk) [3]. As 75% of adult mental health disorders begin in childhood, getting help to young people early has important clinical, social, and political implications [4,10]. Of all the various indicators of SES, low household income and low parental education were found to be the strongest predictors of mental health problems among children and adolescents, with greater impact in early childhood [2]. Young people in rural areas are particularly vulnerable; as a higher risk population, they are less likely to seek help due to their social visibility (ie, the perception that they will be seen accessing health services and this will not remain confidential), and they have fewer services available to them [6,11,12]. There is also disparity in the quality and outcomes of psychiatric care for vulnerable populations, which include ethnic minorities, rural communities, and people of low SES [5,12-15].

Traditional approaches to translating evidence-based interventions into practice have failed to substantially close the gaps in service quality or reduce disparities [16-18]. Alegria et al [18] suggested the limited impact of evidence-based interventions in vulnerable communities may be due to not accounting for community and cultural contexts, such as infrastructure realities (eg, lack of staffing or access to services, and community cultural norms), focusing on individuals without using community resources to support implementation, research findings being disseminated primarily through scientific journals rather than directly to communities, and the gold standard for clinical research, the randomized clinical trial, emphasizing internal validity over external validity, or generalizability, and often excluding vulnerable populations [18].

Computerized therapies have been shown to be effective in alleviating depression and anxiety symptoms in adults, adolescents, and children [19-22]. Most of the available programs online are text-based, with limited interactivity, relying on higher levels of literacy. Efficacy studies of computerized CBT (cCBT) have noted issues with user engagement and high attrition of users [19,23-25]. Interestingly, program specific design features are widely considered in human computer interaction research and in the field of serious gaming [26], but have received little attention in studies of cCBT.

Smart, positive, active, realistic, X-factor thoughts, or SPARX, is a cCBT program that uses a bicentric frame of reference [27]; it combines an exocentric virtual therapist to provide observer perception and reflection with an egocentric game component, which immerses the user in participation through accomplishment of a series of tasks. SPARX engages the user in a fantasy-based world, where the user travels to 7 different provinces to undertake CBT-based challenges and to develop skills [28]. A narrator or “Guide” supports the user throughout the program, and provides encouragement and dialogue promoting the idea that depression is treatable and that by making some achievable changes the user will feel better. The efficacy of SPARX has been assessed by a randomized control trial with New Zealand youth aged 12-19 years accessing help for their depression from primary health care sites [28]. SPARX was shown to be at least as good as usual care (primarily counseling delivered face-to-face with a mental health clinician) [28]. Youth trialing the SPARX intervention also reported a high level of satisfaction and engagement with the program [29,30].

This research project was undertaken as a first step in exploring the “translation” of a computer program developed for New Zealand youth, for use by young people in rural Australia. Issues such as accents and local differences in health care access might mean adjustments to programs are required. Given that (un)acceptability of Web-based programs is a significant factor in attenuating the effectiveness of services delivered in real world settings as compared with efficacy shown in clinical trials it is an important component to assess for methodological and pragmatic reasons [31]. While there is no one definition of acceptability, common sources of information about acceptability, include take-up and dropout rates, reasons for dropout, and patient attitude and satisfaction toward an intervention [31]. Given the exploratory nature of this study acceptability was assessed in terms of general expressions of interest by participants in the use of the program in its current form for self or other, and the suitability of the mode of delivery. This is of interest as issues of dissemination of computerized programs outside of the group that they were designed for are seldom explicitly considered.

## Methods

### Geographical Selection

To engage with the community to assess the acceptability of the SPARX cCBT program (look, sound, and feel) to Australian youth, a naturalistic ontological view was adopted. A naturalistic, or subjective ontology, considers the researcher part of the same experience as the research participants, not discrete from the inquiry. The nature of the research is exploratory; the researcher seeks to understand the reality, actions, and perceptions of individual participants. This is not assumed to be value-free, but interpreted by the researcher [31], an approach consistent with understanding the perspective of local youth on the SPARX program and the need for any visual or verbal components of the program to be “reskinned” (ie, characters, sound, or language in the program changed from the New Zealand context, which includes a range of Maori, Pacifica, Asia, and Anglo ethnicities, to be more relevant to young people living in northwest Tasmania).

A decision to adapt the program would incur cost, thus evidence to support the decision required a degree of impartiality by the researcher. On this basis, a positivist epistemology was applied. Positivist epistemology asserts the researcher has a priori relationships with phenomena, which may be identified and tested by logic and deduction [32]. That is, outcomes may be hypothesized and tested based on predicted causality. The positivist view assumes a value-free and unbiased researcher. Where it is accepted, information systems are inherently a relationship between technology and the social world, value-neutrality cannot entirely exist. Instead, it is argued that paradigms should not be rigid and fixed, but should allow different methods, which are appropriate for the different situations [33,34]. By adopting a less predefined and circumscribed stance, the positivist researcher is open to discovering and understanding nondeterministic variables, which is considered a more valuable inquiry of social systems [34].

As only a small window into local youth opinion was required, both to suit the nature of the inquiry and the way young people were most likely to interact, short focus groups were selected as the primary data collection method, supported by demographic information, observer notes, and content analysis.

One of the small rural towns of northwest Tasmania was selected for the study, which is characteristic of the vulnerable, small communities described. There is one General Practice in the town, no resident psychological service, with a visiting mental health practitioner hosted 1 day per week at a local organization. The nearest regional town with psychology services is over 80 km (50 miles) away; the nearest Headspace office (National Youth Mental Health Foundation offering specialized mental health services for 12- to 25-year olds) is over 200 km (125 miles) away. According to the 2011 Australian Bureau of Statistics Census data [35], the town has a population of 3935, 14% of whom identified as being of Aboriginal or Torres Strait Island origin (compared with a national average of 2.5%), and 15% are aged between 10- and 19-years old. The median weekly household income is AUS$893.00 (5.8% less than the Tasmanian median of $948.00 and 27.6% less than the Australian median of $1234.00). Only 39% of the towns’ population over 15-years old has a post school qualification, compared with 55% of the Australian population.

The two community-based organizations in the town offering a variety of community programs aimed at improving the towns’ health and well-being supported this study. These organizations focus on early intervention and preventative measures to encourage and enhance healthy life and seek to improve quality of life and wellbeing for individuals and families. A range of services are available, including mental health support, health promotion and education, and youth health. One organization supports all persons in the community regardless of their social, mental, or physical condition and generally operates as a self-referral service, although troubled youth are steered to these services through school programs. The other organization specifically targets services to meet the needs of the local Aboriginal community, but also includes referrals from police to youth prevention and diversion programs. The visiting mental health practitioner is hosted by this organization through a Medical Specialist Outreach Assistance Program. As such, these organizations do not represent the broader community of young people, but provide a more purposive sample for assessing an adolescent mental health intervention by means of a focus group interview. Ethics approval for conducting this research was obtained from the Tasmanian Social Sciences Human Research Ethics Committee.

### Recruitment

Both organizations preferred to recruit potential participants themselves, and arrange groups to optimize dynamics based on their knowledge of the individuals. Three participants were recruited from one of the organizations, 2 males and 1 female (these participants did not want to participate in any of the second community organization groups because they did not feel comfortable in an alternative setting. They also wished to have gender-specific groups). The research team determined it was important to capture the views of marginalized individuals and we were, therefore, keen to accommodate these preferences. Hence, semistructured interviews were carried out with the 2 male and 1 female participants.

The second organization recruited 10 males and 3 females. Again, this organization’s staff felt the individuals would best participate in groups according to gender, and organized their participants into a younger male group (with participants 12- to 15-years old), an older male group (with participants 16- to 18-years old), and a group for females.

### Conducting the Focus Groups

The focus groups were held at the participating organizations in consecutive weeks, with an information session held the day before each focus group. At the beginning of the focus group sessions participants were asked to record their age, gender, ethnicity, and whether or not they played computer games.

**Figure 1:**
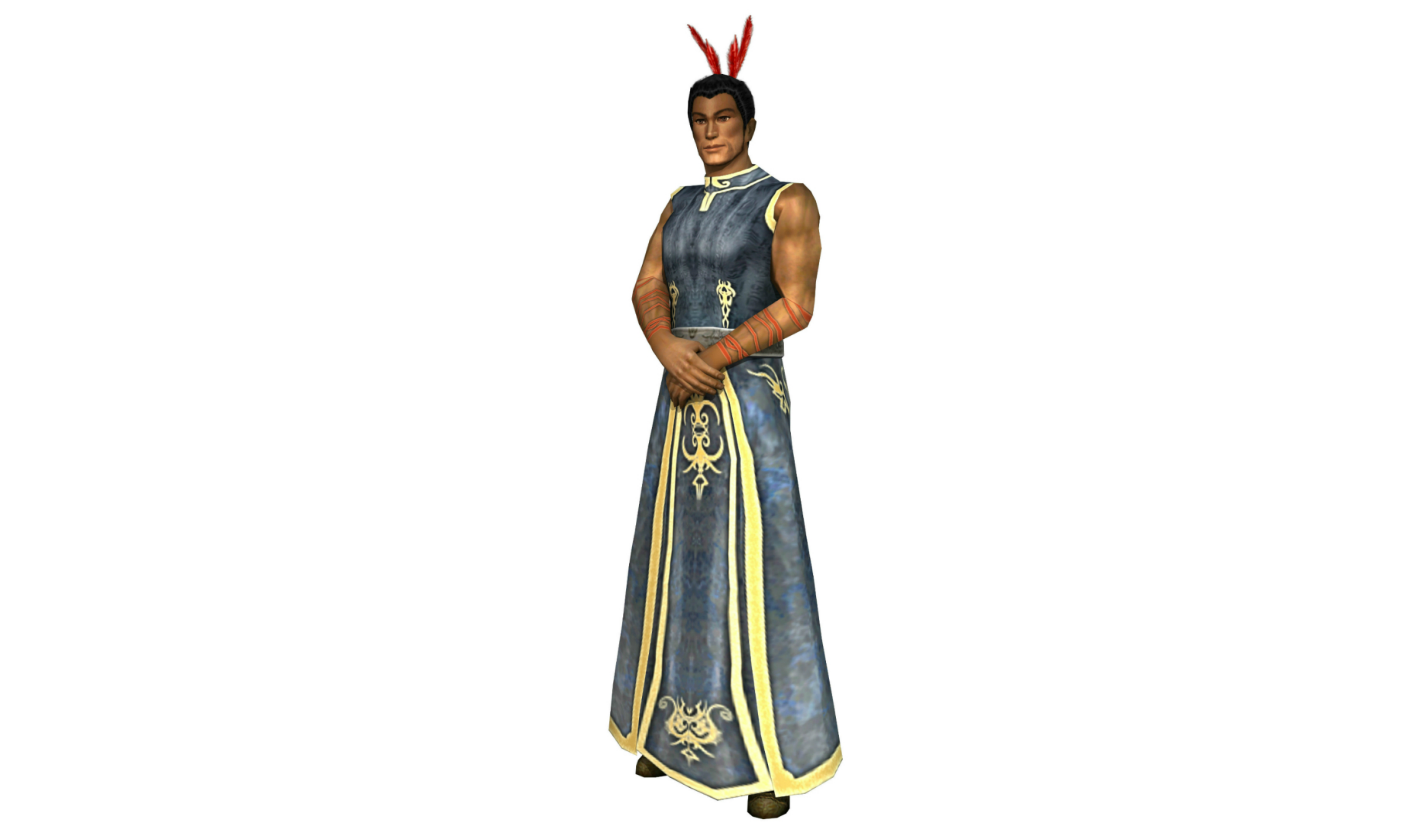
SPARX guide.

**Figure 2:**
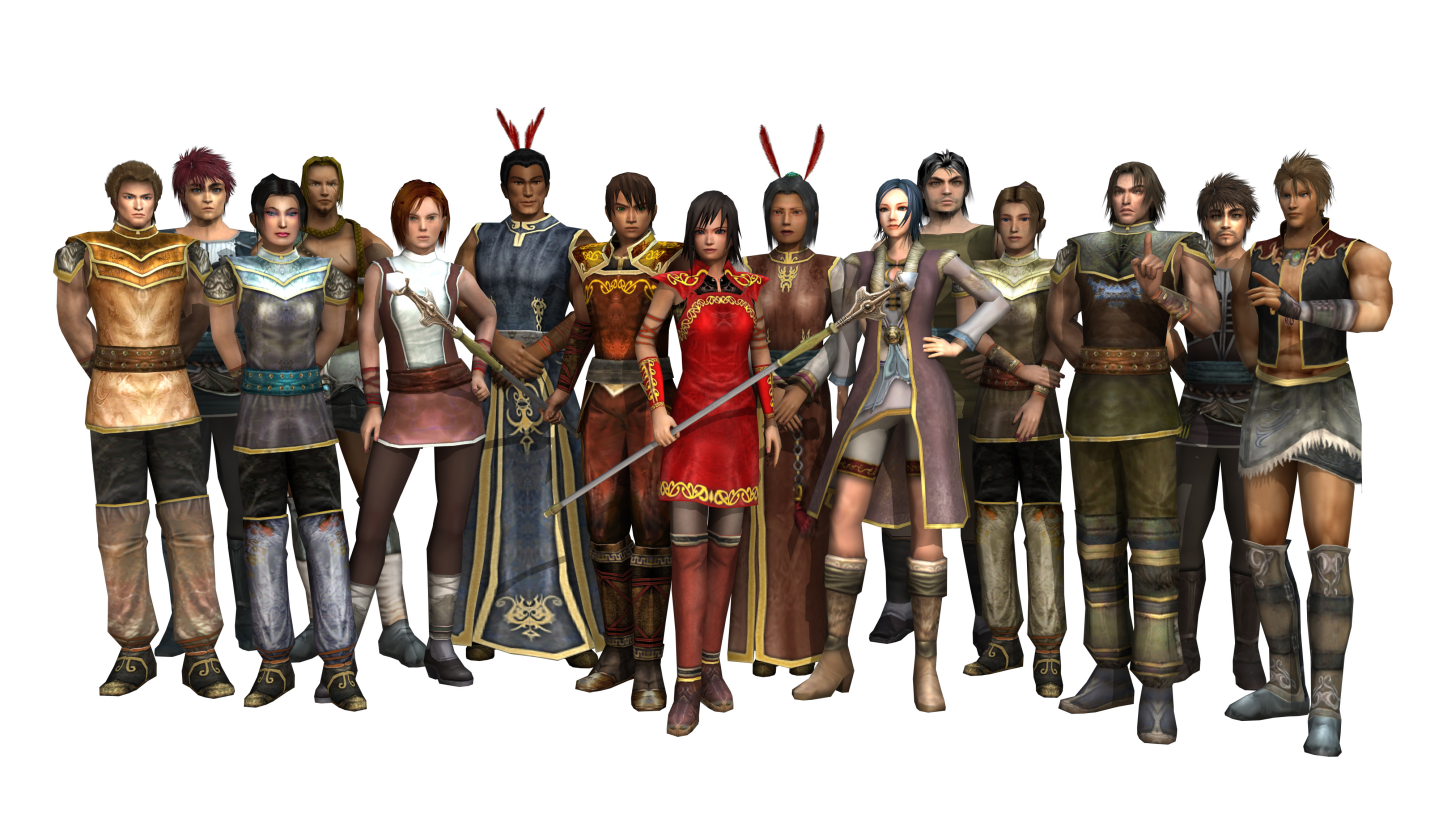
All SPARX characters.

The SPARX program was introduced by using a short (5 minute) video trailer of the program. The trailer included audio of the Guide character supporting a program user, avatar customization, and the visual effects of being transported into the SPARX fantasy world (Figures 1 and 2). Following this, three PowerPoint slides were shown, which illustrated 4 of the program’s “provinces”, the tasks to be completed in those provinces were described, and the personal journal/user notebook was demonstrated. The focus group was also asked a list of questions to help evaluate SPARX, outlined in Textbox 1.

The clinical psychologist of the research team (HB) acted as the Facilitator, while another member played the role of Observer (CC). Neither were known to participants prior to the information sessions.

**Predefined questions to initiate open-ended discussion.**

1. What do you think of the way SPARX looks?

2. What do you think of the way SPARX sounds?

3. Do you think young people around this area might be interested in using the program? If so, why? If not, what about if they were feeling down or depressed?

4. If young people were to use SPARX, where might they use it (eg, at home, school, etc)?

5. What do you think might get in the way of young people using SPARX?

6. Do you think any changes need to be made to SPARX so that young people would like to use it? If so what could be changed? How important would it be to make these changes?

7. What do you think about the use of the word “Depression”?

### Analysis

As this was a small study, a hybrid approach in developing a data-driven code was followed as described by Boyatzis [36]. In the first stage all interviews were used in the development of the themes and coding framework, negating the need for sampling to model coding. In the second stage, our own theories (CC, HB) were used as a means to articulate meaningful themes, replacing comparison of themes across samples. Coding the rest of the raw data was not required as the interviews were not sampled.

Thus, to systematically conduct thematic analysis, the procedure followed in this study was in two stages:

1. Identification of the dependent variable and use of all 5 focus groups/interviews
2. Development of themes and codes
  - Reduction of the raw data
  - Identifying themes within samples
  - Comparison of themes to own theories and research literature
  - Creation of a code
  - Checking reliability by revision and refinement of category system

Audio recordings of the interviews were transcribed using Olympus Sonority v1.2.0 voice recording software. The transcripts were entered into Microsoft Excel 2010 software for organization, identifying the group, gender, and age of participants. Observer notes and content analysis were added at the appropriate questions. Blocks of text were summarized according to the specific interview question and research aim (Table 1). Transcripts were then compared and similarly themed summary text was grouped (Table 2).

Emerging themes were identified through repetition of a common word or action, for example “privacy” or “using it in private/at home/where no one else can see”. The views of gamers were subsequently compared with nongamers to understand how a prior interest in computer gaming might affect their view of SPARX, and within the context of the personal sphere of participation for meaningful use of technology in health [37].

Sixteen codes were generated. To identify categories, an iterative grouping process was used where the codes were at times split, and regrouped with indicators and text fragments as examples to find where they best represented the themes and data within, and to select category labels that best represented these. Once these categories were established as nodes and subnodes within nVivo software, the transcripts were recoded according to this framework by members of the research team individually (CC, HB). This double-coding technique sought to test the reliability of the coding framework. Reports were compared and reviewed collectively to discuss and resolve differences. The revised final category system, themes, and transcripts were reviewed (TF) and found to be similar to information derived from SPARX-related focus groups previously conducted with young people in alternative education programs [30].

**Table 1:** Excerpt of summarized block of text from focus group 1 interview in Microsoft Excel (boys, gamers, age 13).

Line	Speaker	Quote	Field notes	Summarized text
10	R	*What about how the characters sound. Any feedback about that?*		
11	Y1	*Well, one of them sounded a bit like a Kiwi*		The New Zealand (Kiwi) accent was identified by 1 of the 5 participants, but in general it was not thought to be important by the group.
12	R	*A bit like a Kiwi. Did anyone else notice that?*		
13	Y2	*No*		
14	Y3	*No*		
15	Y4	*Na*		
16	Y5	*Na*	1:5^a^	
17	R	*OK. Do you think other people would notice that they sound a bit like a Kiwi?*		
18	Y1	*Probably, I don’t know*		
19	R	*Do you think it would bug people?*		
20	Y1	*No*	*Same person who identified Kiwi accent*	
21	Y2	*Not really*		
22	R	*Not really?*		
23	Y3	*It’s not very important*		

^a^1 out of 5 participants noticed one voice actor sounded “Kiwi”.

**Table 2:** Groups of similarly themed summary text.

Group	How does the program sound?
1	Five of 6 participants said it sounded like it was for an audience of Grade 7-8 (12-14 y); having text displayed as well as the audio was helpful.
2	The New Zealand (Kiwi) accent was identified by 1 of 5 participants but in general it was not thought to be important by the group.
3	The group thought it sounded okay and were happy with the voices. They did not think any changes needed to be made. If it was to be “Australianized”, 1 participant (nongamer) thought an Australian accent would be more attractive to local youth, but if the choice was between not having the program or having the program with the current accents all 3 would choose to have the program as it is
4	Thought it sounded “Cool”
5	Thought it was “OK”

## Results

### Demographics

Five focus group/interview sessions were held with a total of 16 participants, (12 male and 4 female) between the ages of 13- and 18-years old. Four participants reported their ethnicity as Aboriginal, 5 as Australian, while 7 reported none or left this field blank. Thirteen of the 16 participants identified themselves as a computer gamer (Table 3).

The categories derived from thematic analysis of the data were personalization, engagement, and stigma.

**Table 3:** Participant self-reported demographics.

Group/session	N	Gender		Age						Ethnicity			Computer Gamer	
		M	F	13	14	15	16	17	18	Aboriginal	Australian	None	Yes	No
1	4	4		4								4	4	
2	6	6				2	1	2	1	1	5		6	
3	3		3	1	2					3			2	1
4	1		1		1							1	1	
5	2	2			1		1					2		2
Total	16	12	4	5	4	2	2	2	1	4	5	7	13	3

### Personalization

Having options to make personal choices was consistently valued across all groups with all participants, “because everyone’s really different*.*” [Male, 15- to 18-years old]

Table 4 outlines some of the ways choice was valued and examples of how this was expressed.

Additionally, the participants accepted that while some people would value being able to use the program in private, without telling anyone, others would prefer to use it with a counselor, to augment counseling sessions, or to use the program in a group therapy session, “It’d probably be like a bit of a first step, like they go through that [SPARX], then they they’d go to a counselor sort of thing.” [Male, 15- to 18-years old]

The personalization of the program itself was also seen to be important. This was strongly represented in comments relating to the choice of gender of the avatar, with both males and females suggesting making the gender of the guide a choice for users. The importance of this choice was not just for aesthetics, but because the young person might not relate well to the gender seen as being the cause of, or contributing to, the young person’s issues in real life.

The ability to recommend this program to friends who might not otherwise agree to see someone for help was also valued by the groups.

**Table 4:** Valued choices on ways to get help and supporting quotes.

Choice	Quote
1. Choose how they got help, who to tell, or choosing not to tell or be reliant on anyone in order to get help	*They don’t really have to talk to an actual person about it, and that way they don’t have to worry about getting judged with the feedback and stuff like that.* [Male, 15- to 18-years old] *Brothers and sisters mightn’t know that you’re feeling that way, and you may not want them to know.* [Male, 13-years old]
2. Choosing when and where they could use the program	*You know… go home and play it all night all day and stuff.* [Female, 14-years old]
3. Being able to get help outside of a counseling session	*If they have a counselor or someone like that, they can recommend it if they don’t see them very often. Because say, like once a week and the person doesn’t feel like that’s enough, they could go to this program.* [Male, 15- to 18-years old]

### Engagement

This theme referred to young people’s access to and use of computers in general and their acceptance of the SPARX computer game as a tool to deliver health care. All participants used computers, and some specifically identified they used computers when they were feeling down. Participants either identified as people who usually played computer games or not. Most (13 of 16 participants) played computer games. Those who played computer games were very accepting of this program:

It's just cool… it's a different way... because you know, you go to a counselor and stuff and they have all these different ways of doing things but like, nobody’s ever really thought of a computer game or something. It's usually like “tell me how you're feeling”, or “write it down” and stuff, but not “play it”.

Female, 14-years old

Those who did not play computer games said they would be less likely to engage with the SPARX program. One nongamer was quite vocal within her group and open about her lack of interest in this medium as a way of getting help*,* “To be honest, I hate computer games. Some people like me—I wouldn't want to play a game.” [Female, 13- to 14-years old]

This allowed comparison of her responses to others. For example, her view of a fantasy-based genre was different than others, which is outlined in Table 5.

**Table 5:** Female responses to game genre.

Speaker	Quote
Researcher	*If you were looking for help would you want fantasy or real?*
Group 3 female nongamer	*Modern*
Group 3 female gamer	*Fantasy - I think that look is really cool*
Group 4 female gamer	*It's really cool and it’s cool how you meet different people, like the travelers pop up and stuff*

While some recognized that the Guide had a New Zealand (“Kiwi”) accent, they also stated that this was not important (Table 2). The most consistent response to the question about proposed changes for the program was to provide an option to change the gender of the Guide. The importance of this change, when offered on a scale of 0-10, with 0 being not at all important, and 10 being very important, ranged from 4-8. When asked if it was more important to make these changes or make the program available as is, all were emphatic that it was more important to “get it out there.” [Female, 13- to 14-years old]

School was not favored by any group as a place to use SPARX for fear of school peers finding out that a young person was having personal problems and reacting in a negative way. The fear of reprisal was so large some participants stated they would not stop to read posters advertising the program around their school because of its focus on mental health-related issues:

Not a lot of people would want to stop there, if it was something like that [SPARX]. Someone will say, “Why are you looking at that?”, “Why do you need that?” There’s a lot of bullying around.

Female, 13- to 14-years old

While most reported that they would use SPARX in the privacy of their own home:

Yeah because if it’s fully online – people don’t like people knowing about their problems, so they’ll be judged. So we’d do it in private [at home].

Male, 15- to 18-years old

Some identified alternative safe places in the community:

The Online center; somewhere like that but with a bit more privacy.

Male, 13-years old

Females stated that boys did not like to talk explicitly about their feelings, so this medium potentially offered them an alternative way to explore their feelings safely without having to talk to others, and get the help they needed (perhaps without even realizing it). Boys did comment that some people didn’t like talking about their problems, and that the program would be easier than going to a counselor. While the cCBT medium allowed users to get help without having to divulge their thoughts and feelings to others, the narrative of the game and use of different characters was identified as providing an element of socialization and “life” to the program:

I like how you've got that Guide and that you can personalize yourself. Like the journal thing - that's pretty cool.

Male, 15- to 18-years old

Table 6 outlines three of the reasons why participants were interested in receiving help through a computer game-like medium.

**Table 6:** The reasons why participants were interested in receiving help through a game-like medium, and examples of how this was expressed.

Reason	Example Quote
1. It is a medium that is known and accessible to them	*Lots of people play games on the computers and in gaming systems.* [Female, 13- to 14-years old]
2. They use computers anyway to feel better	*When I’m feeling a bit sad or down, I just hop on the computer really.* [Male, 15- to 18-years old]
3. It is a nonthreatening way of getting help	*A lot more fun for them too...easier for them to deal with whatever's going on through a game.* [Female, 13- to 14-years old]

### Stigma

The stigma of depression and fear of being judged negatively by others was very apparent across all groups/sessions in this study. It was described by participants as the reason young people do not accept they have symptoms and need help, and as preventing them from asking for assistance:

Some people don’t like to talk about [depression or mental health issues], to people about their problems. It’s too embarrassing… like they’re useless.

Male, 15- to 18-years old

Participants also commented that health professionals were sometimes reluctant to accept a young person had a problem and needed help, further limiting the young person’s confidence in asking for help*,* “Because different professionals sometimes, sometimes they’re in denial, because they don’t think you’re depressed at all.” [Male, 15- to 18-years old]

The effect of stigma and their visibility in a small town was evident at a manifest and latent level. In response to a comment that there were free counselors available, a participant commented*,* “That doesn’t always help.” [Male, 13-years old]

Another participant related her experience of using online chat sites instead of using local practitioners, “because it's not in the town where everyone can just tell everyone.” [Female, 14-years old]

## Discussion

### Principal Findings

In this exploratory focus-group study, we found that participants wanted help for mental health issues, but there is a stronger need to have choices available to them and to control how they access services. They considered protecting their privacy and visibility in their small community to be paramount. They saw online therapy as providing a number of features that promise this control, getting help without having to tell anyone, and getting help without physically seeing a person or attending a place known in the community. Participants considered the SPARX cCBT program to be appealing and engaging to young people. Design features such as the personalization of an avatar, the socialization with the Guide and other characters, journaling, and the use of encouraging feedback were seen as engaging and helpful. Despite the fact that it was not an Australian program it was considered to be appropriate for local youth. SPARX, in a “playful” medium and using a fantasy-based genre, is a format like other computer games, which many young people like to play and are likely with which to have a level of competence. In this format, young people are accepting of differences in the look and sound of characters.

Previous research has identified that young people often do not seek help from health providers for mental health concerns even if they are concerned about these issues [38-40]. The present findings underscore that rural youth wish to have choices, autonomy, control, and privacy in accessing mental health services. They view Web-based resources as an important opportunity to address this.

It is of interest that young people wished to be able to use the program in private or to be able to use it with a counselor or in a group. In a domain in which an empathetic and supportive relationship with a counselor is a mainstay of therapy, computerized therapies have been regarded warily by practitioners [2,41,42]. In a 2013 study of Australian rural clinician views on using computerized mental health therapies, while most supported the concept, models in which programs were used in conjunction with traditional face-to-face therapy were preferred [42]. Youth in the present study included this model as one of several ways the program should be available. However, they also considered that alternative methods of access should be offered to reflect the different ways people prefer to get help. This is consistent with findings from Fleming et al [30] when exploring the views of young people alienated from mainstream education. The SPARX trial in New Zealand demonstrated that SPARX can be effective with minimal input by a clinician as only research assistants were involved [28].

School is considered an important part of the social life of young Australians; playing a key role in providing help services and the opportunity to use select interventions with extensive reach [12]. The views of participants in this research, however, suggest local schools are not a safe place for them to be openly receptive to communication or interventions for mental health issues. This not only indicates the stigma of depression in the community remains high, but also raises an issue about how to disseminate information on available programs. Restricting access to online therapies for use only in conjunction with clinicians or at school may limit its potential and perpetuate young peoples’ reluctance to seek help.

The finding that the SPARX program was appealing is consistent with the popularity of computer games using a fantasy-based genre, which has been reported to comprise over 80% of the gaming market [43,44]. However, it is important to note that SPARX is not a computer game and is unlikely to be considered appealing to a general audience alongside other computer games. Rather, SPARX is a therapeutic tool, which has used a playful interface to increase engagement with young people. User attrition has been an issue in trials of many self-help programs [23-25]. Aspects of appealing design and engaging program features are worthy of explicit consideration. It was of interest that the New Zealand accents and graphics in SPARX did not affect the acceptance of the program in this rural Australian population. All considered, having SPARX available is more important than any changes that might customize it to local culture. In fact, if there were to be changes to SPARX, other changes (eg, being able to choose a male or female guide) were seen as more important than regional accents.

### Strengths and Limitations

Where community participation is considered important, introducing some bias may be a trade-off. While participants were not selected on the basis of any objective mood measure, the youth workers did know the individual participants better than the research team, and as such were able to seek out a purposive sample. Most participants said they had felt down in recent weeks. As a step in translating evidence-based therapy into practice, it relies on finding evidence of meaningful use and advocating value from the perspective of vulnerable groups, rather than performing dispassionate science. Nevertheless, quantitative measures were used to support or refute interpretations, and accepted methods of systematic process and analysis were employed and explained.

The small sample size and single-community setting for this research makes this study just a small window into the views of local youth; the focus groups were exploratory and as such the findings of this research do not profess generalizability or offer authority.

### Implications

This research has demonstrated that a proven tool can be adopted when the context has changed. Limitations such as accent have little effect when the medium is known and the tool has high fidelity. Young people like computer games. This playful medium offers a nonthreatening way to explore identity and express feelings, in a format that is hugely popular and could overcome literacy barriers and cultural specificity through narration and fantasy-based worlds.

In spite of recent investment in local Internet infrastructure, in 2011 only 65% of northwest Tasmanian dwellings were connected to the Internet, falling to 62% in some west coast towns, in comparison with 74% of Australia [35]. While youth indicated a preference to use this type of program at home, more safe places must be found as part of community implementation. Other options offered by participants included the Internet access center or youth centers, but safe places are likely to vary between communities.

### Conclusions

Adolescents have strong views about the dissemination and content of mental health treatments and these views should be considered in the development of services. Computerized CBT therapy offered with and without therapist support and via settings other than school was seen by young people as a promising way to increase access to treatment for rural and remote youth. The game-like interface offered by the SPARX cCBT program was appealing to Tasmanian youth and making the program available was seen as more important than making any modifications to it. It may be an interesting coincidence that fantasy-based computer games have world-wide appeal, but it is an exciting prospect for expanding the reach of evidence-based therapies to more communities.

## Abbreviations

CBT: cognitive behavior therapy
cCBT: computerized cognitive behavior therapy
SES: socioeconomic status
SPARX: smart, positive, active, realistic, X-factor thoughts
